# Metallic: A Bivalent Ambimodal Material Property?

**DOI:** 10.1177/20416695211037710

**Published:** 2021-09-14

**Authors:** Charles Spence, Fabiana M. Carvalho, David Howes

**Affiliations:** Crossmodal Research Laboratory, University of Oxford, Oxford, United Kingdom; Department of Food and Nutrition, School of Food Engineering, University of Campinas, Campinas, Brazil; Centre for Sensory Studies, Concordia University, Montreal, Quebec, Canada

**Keywords:** metallic, taste, flavour, odour, material perception, ambimodal

## Abstract

Many metallic visual stimuli, especially the so-called precious metals, have long had a rich symbolic meaning for humans. Intriguingly, however, while *metallic* is used to describe sensations associated with pretty much every sensory modality, the descriptor is normally positively valenced in the case of vision while typically being negatively valenced in the case of those metallic sensations that are elicited by the stimulation of the chemical senses. In fact, outside the visual modality, *metallic* would often appear to be used to describe those sensations that are unfamiliar and unpleasant as much as to refer to any identifiable perceptual quality (or attribute). In this review, we assess those sensory stimuli that people choose to refer to as metallic, summarising the multiple, often symbolic, meanings of (especially precious) metals. The evidence of positively valenced *sensation transference* from metallic serviceware (e.g., plates, cups, and cutlery) to the food and drink with which it comes into contact is also reviewed.

## Introduction

*Metallic* is a term that is used to describe sensations from pretty much every sensory modality. As such, one might legitimately want to consider whether it is, in some sense, an amodal (e.g., Lewkowicz & Turkewitz, 1980) or *ambimodal* (as will be suggested here)^1^ stimulus property, despite rarely/never being mentioned as such in the literature. At the same time, however, one of the most intriguing features of those sensations that people choose to describe as metallic is that the valence attached varies dramatically as a function of the sensory modality to which the term is applied. While metallic visual impressions tend to be positively valenced (i.e., pleasant and attractive), they are mostly negative when referred to in the context of stimulation by (or involving) the chemical senses (see Reith & Spence, 2020, for a recent review). The shiny visual surface properties of certain metals have long appealed, especially in the case of precious metals such as silver and gold (e.g., Aldersey-Williams, 2011).^2^ In part, this may reflect the symbolic meaning that is so often attached to metallic stimuli, especially precious metals (Chadwick, 2021; see also Karana, 2010; Pye, 1978; The Silver Spoon, 1997). This is presumably due to a combination of their rarity, their highly appealing visual qualities (Anderson, 2011; Kirchner et al., 2007; Matsumoto et al., 2016), and their durability/malleability.

Apart from their preciousness, metals are so foundational to civilisation that whole epochs are distinguished by reference to them: Bronze Age, Iron Age, Age of Steel, and so on (Miodownik, 2014). People associated with working metal are regarded with some ambivalence, however, as in the Biblical story of Cain (the first blacksmith) and Abel. Indeed, metalsmiths often have an ambiguous status: Their craft may be vital, but they are perceived as “dirty” (e.g., see van Beek, 1992).

The descriptor *metallic* is, rightly or wrongly (as we shall see later), also used to describe sounds, smells, tastes, and even the feel of objects, materials, and surfaces. For instance, as regards sounds, Hermann von Helmholtz published his *On the Sensation of Tone as a Physiological Basis for the Theory of Music* in 1862 as an attempt to account for the perception of tone quality. Helmholtz determined that the quality of a musical tone depends on the form of vibration (called Helmholtz Motion) as well as the strength and number of the harmonic upper partial tones. He described the general characteristic of what is called “a *metallic* quality of tone as the comparatively continuous and uniform maintenance of higher upper partial tones” (Helmholtz, 1877, p. 145, italics in the original). However, a higher number and maintenance of upper partial tones (or overtones) is undesirable. Helmholtz (1877, p. 145) states that the quality of tone made by wood instruments in which the overtones die away rapidly is perhaps more satisfactory to a musical ear than the harmonicons formed of steel rods or plates with their piercing inharmonic upper partial tones.^3^

The sound of a material depends on well-defined physical parameters of the material itself, namely, the density and the elastic modulus, as well as the form of the object used to produce the sound (Laughlin & Howes, 2014). Keeping the object’s form constant (e.g., a tuning fork or a bell) and changing the density or elastic modulus of the material will change the pitch of the note produced. Ashby and Johnson (2002) developed a multidimensional scaling map of acoustic properties of a wide range of materials by plotting the acoustic pitch against acoustic brightness. Acoustic brightness quantifies how much a material absorbs sounds. Bright materials, like glass, emit sounds for a long time whereas dull materials, like foams, absorb sound strongly. The multidimensional scaling map shows that materials from the same family or group (e.g., polymer, metal) tend to cluster together due to the correlation of acoustic properties. The quality of the “metallic” sound produced by the metals as a group is experienced as a note of a high pitch (frequency) with high brightness (a factor combining duration and amplitude; Ashby & Johnson, 2002, p. 72). Meanwhile, in the case of orchestral music, people talk of the brass family for the *pure* metallic sounds (that said, there is a hint of metal in the sound of violin strings as well, and once again, this is typically described as something that should be avoided; Mesli, 2016).^4^ Those suffering from tinnitus often describe the sound they hear as tinny (Meyer, 2013; RSS Print, 2020). Meanwhile, sounds described as *metallic* have also been recorded in the Mariana Trench Marine National Monument. According to researchers, the latter sounds are thought to be produced by the baleen whale (Nieukirk et al., 2016).

Many metals give off a somewhat distinctive sound when struck (e.g., Giordano & McAdams, 2006; Kunkler-Peck & Turvey, 2000; see Spence, 2020c; Spence & Zampini, 2006, for reviews of material properties that are conveyed by, or can be discriminated on the basis of, material-striking sounds) or rubbed. In the latter category, consider only the distinctive sounds given off by Tibetan metal singing bowls. These metal alloy bowls were traditionally used by Himalayan monks in ceremonial/meditative situations (Inácio et al., 2006; Perry, 2014; Terwagne & Bush, 2011). It has long been suggested that listening to/meditating on the sound made by these bowls when rubbed may have a therapeutic role, though robust evidence in support of such a claim has yet to be published (Goldsby et al., 2017; Stanhope & Weinstein, 2020). However, once again, the pleasant sound (of metal) is typically not described as *metallic.* Here, it would be interesting to know whether the sonic properties of mercury (the only metal that behaves as a liquid at room temperature) are also qualitatively distinctive from the sounds of other liquids, such as water or wine when poured (cf. Decremps et al., 2009; Velasco et al., 2013; see also Spence, 2020d).

The particular configuration of tactile/haptic properties shared by many metals, in terms of their specific density, hardness, and thermal diffusivity (Bergmann Tiest & Kappers, 2009), is likely to be pretty distinctive when people handle metals too (Baumgartner et al., 2013; Bergmann Tiest, 2010; Ingvarsdóttir & Balkenius, 2020),^5^ even in the absence of any cues from the normally dominant visual modality (Hutmacher, 2019). Indeed, it has been suggested that the weight of metallic cutlery may be an important part of its appeal (see Michel et al., 2015; Piqueras-Fiszman & Spence, 2011; though see also Harrar & Spence, 2013),^6^ together with the fact that contemporary metallic cutlery (i.e., stainless steel or silver) does not appear to convey a noticeable taint to the taste/flavour of food. Using a lignum vitae mallet apparently feels almost as if one is swinging a steel hammer (on the sound of mallets, see Freed, 1990). The surface of an ironwood feels warm (not cold and metallic) to the touch but lignum vitae feels like steel when it is wielded. Note that lignum vitae is the hardest of commercial woods according to the Janka scale.

Perhaps the most interesting use of the term *metallic* is to describe those sensations that happen to be associated with the stimulation of the chemical senses. Here, though, there is seemingly ongoing uncertainty as to whether metallic should be considered as a basic taste (as Wundt believed back in the 1880s; see also Fincks, 1886, p. 682; see Bartoshuk, 1978, for a review), or an olfactory sensation (Epke & Lawless, 2007; Lubran et al., 2005), and, if so, whether it is one that is differentially associated with the orthonasal versus retronasal routes (see Rozin, 1982; Wilson, 2021, on the distinction between these two *ways* of smelling). Some researchers have even described metallic sensations as flavours too (e.g., see Hunziker et al., 1929; Pirkwieser et al., 2021; Stark & Forss, 1962; see also Vasilaki et al., 2021).^7^ The kind of sensation that is experienced, so it turns out, may depend on the particular metal (or metal salt) that happens to give rise to the sensation, though all three routes (gustatory, orthonasal, and retronasal/flavour) have received empirical support (Cohen et al., 1960; Cuppett et al., 2006; Epke et al., 2009; Hettinger et al., 1990; Lawless, Rapacki, et al., 2003; Lim & Lawless, 2005, 2006; Omur-Ozbek & Dietrich, 2011; Schiffman, 2000; Skinner et al., 2017; D. A. Stevens et al., 2006; Zacarias et al., 2001).^8^ At the same time, however, it is also worth stressing that many metals have no discernible taste, aroma, or flavour (such as, for example, in the case of aluminium foil).

Electrical stimulation of the tongue (known as electrogustometry) may give rise to a metallic sensation (Tomiyama et al., 1971). This has led some researchers to argue this as a sensation that is not captured by the other basic taste terms (e.g., Tomita et al., 1986; though see also Ajdukovic, 1984, 1990; Bujas, 1971). Perhaps unsurprisingly, electrically induced metallic tastes are not reduced in intensity by closure of the nose, suggesting that the induced sensation is more akin to a basic taste than a retronasal smell or flavour (Lawless et al., 2004, 2005). That said, one might want to question quite what the relevance of the delivery of such unnatural electrically induced taste stimuli is, at least as far as establishing the fundamental nature of metallic sensations is concerned (Frank & Smith, 1991; cf. Keeley, 2002).

In the past, researchers interested in assessing basic tastes have sometimes resorted to the use of crossmodal correspondences and other comparisons/classifications of stimulus similarity/association (e.g., O’Mahony, 1983). Looking to the future, there is likely to be an intriguing line of research relevant to establishing the crossmodal correspondences that people have with metallic sensations. And, should *brassy* be accepted as a descriptor of a class of metallic sounds, then crossmodal correspondences have already been established with the basic tastes, with, for example, the latter being more strongly associated with bitter-tasting foods than with sweet, sour, or salty foods (see Crisinel & Spence, 2010, 2012).

### Metallic Notes Experienced in Food/Drink

On occasion, there have been consumer reports of metallic sensations being detected in food and drink (Pirkwieser et al., 2021). However, this attribute is nearly always negatively valenced (meaning that it is normally perceived, or considered, as a fault or off-taint). One of the first reports of a metallic taint to have appeared in the literature was published almost a century ago in milk samples (Hunziker et al., 1929; see also Bodyfelt et al., 1988; Heiler & Schieberle, 1997; Swoboda & Peers, 1977). Sjöström and Cairncross (1955) also describe the unpleasant metallic taint in old carrots, in cooked spinach, and in boiled summer squash. Traditionally, a metallic sensation has also been associated with canned tomatoes (Malmendal et al., 2011 see also Zacharias & Tuorila, 1979). Intriguingly, however, consumers apparently got used to this particular metallic taint, with some even complaining when improvements in packaging design eventually eliminated this *fault*, or taint (Rosenbaum, 1979, p. 81). One also finds metallic characteristics reported in the assessment of various species of freshwater fish (Chambers & Robel, 1993), not to mention metallic sensations appearing in the panel rating of fresh red meat (Mitterer-Daltoe et al., 2012). Metallic is sometimes also mentioned as a sensory descriptor in the evaluation of coffee (Bicho et al., 2012), and occasionally in the dysguesia that has come to be known as *pine nut syndrome* (Ballin, 2012). High Unique Manuka Factor honey is occasionally described as having a metallic taste as well (cf. Sarheed & Debe, 2020). And finally here, a number of artificial sweeteners (and salts) also have a metallic taste (Riera et al., 2007; Schiffman et al., 1979, 1985). One of the side effects of certain antidepressants is a metallic taste (Doty et al., 2008). A metallic taste has also been reported as a side effect of certain local anaesthetics, such as, for example, Bupivacaine (Lo, 1999).

Traditionally, people were often advised to avoid drinking red wine when eating seafood (especially white fish) because of the unpleasant sensation that sometimes resulted (Spence et al., 2017). Reports of a ferrous taste, an unpleasant fishy, and a metallic odour and possibly also bitterness were not uncommon. The suggestion is that the ferrous ion in some older red wines was responsible for the unpleasant aftertaste (Tamura et al., 2009). That said, contemporary winemaking methods/practices have largely eliminated this problem (given the widespread use of stainless steel barrels). Fatty acids are not perceived as fatty; all of them are described as irritants, and some of them (e.g., stearic, linoleic, and linolenic acids) are also perceived as metallic (Delompré et al., 2019).

### Electric Taste Augmentation

In the past few years, there has been a growing interest in the potential benefits of incorporating digital flavour augmentation technologies into traditional eating and drinking experiences. Gaining digital controllability over the gustatory sense is one of the fundamental aspects when considering immersive media delivering authentic flavour augmentation. Several studies have explored the possibility of using nonchemical stimulation methods to trigger gustatory responses (Bujas, 1971; Lawless et al., 2005; D. A. Stevens et al., 2008). One of these methods is the use of electric devices to stimulate different regions of the tongue in order to assess the qualities and thresholds of the perceived taste sensations. The electric stimulation can be delivered by electrodes attached to the tongue (Karunanayaka et al., 2016; Lawless et al., 2005) or can be added to the food and drink via a utensil (e.g., cutlery, straw, or soup bowl; Nakamura & Miyashita, 2013; Ranasinghe et al., 2019). In some cases, electric utensils have been shown to modulate the perception of the basic tastes associated with foods as they are being consumed (Aruga & Koike, 2015; Ranasinghe et al., 2019).

In addition to reporting enhanced responses to sour and salty (or salty-bitter) tastes, the research also shows that one of the most commonly perceived sensations is the metallic taste (Aruga & Koike, 2015; Lawless et al., 2005; Nakamura & Miyashita, 2013). In fact, the undesirable metallic taste produced by electrical stimulation is one of the key challenges associated to this approach (Ranasinghe et al., 2019). However, the development of methods and technology to enable the sensation of taste as a digital media, which delivers and controls the experience of taste electronically, is still at an early stage.

### Metallic Sensations Among Chemotherapy Patients

Metallic sensations are often reported by patients in a medical context, especially those undergoing chemotherapy (Carson & Gormican, 1977; IJpma et al., 2017; Jensen et al., 2008; Rehwaldt et al., 2009; Turcott et al., 2016), such as, for example, platinum-based chemotherapy (Pirkwieser et al., 2021). Indeed, many patients undergoing chemotherapy report sensations such as a *metallic taste,* a *bad taste,* or the *taste of blood,* among other symptoms. These metallic tastes are also uniformly negatively valenced (see Reith & Spence, 2020, for a recent review). However, that said, it is currently unclear to what extent the metallic sensations that many patients undergoing chemotherapy report (e.g., IJpma et al., 2015), refer to a specific descriptor tied to a particular sensation, or class of sensations, or whether instead the term might actually be used as a general descriptor for any kind of unpleasant mouth sensation, regardless of its phenomenal quality (see Reith & Spence, 2020, for a detailed discussion of this point; see also Ruiz-Ceamanos et al., submitted).

### Metallic as a Descriptor of Unfamiliar and Unpleasant

One finds that a number of other unusual sensations have also been described as metallic. So, for example, astronauts have described space as smelling like: “gunpowder, hot metal, welding” (New York Hall of Science, 2016; cf. Taylor et al., 2020). Environmental exposure to metal fumes has also been reported to give rise to metallic taste sensations (Armstrong et al., 1983). It is intriguing to note how no matter whether it is sound, smell, or taste/flavour, *metallic* is often used in those situations where the sensation is unfamiliar (e.g., New York Hall of Science, 2016; Nieukirk et al., 2016) and often negatively valenced (e.g., Mesli, 2016; Pirkwieser et al., 2021). One of the other peculiar qualities of metallic taste sensations is how they tend to be long-lasting, and not necessarily obviously immediately related to what has been (or is being) consumed, be it in the case of pine nut syndrome (Ballin, 2012) or among those undergoing chemotherapy (Logan et al., 2008; see Reith & Spence, 2020, for a review). Perhaps also worth noting here is how when the stimulation associated with metallic stimuli is pleasant it is rarely described as “metallic”—consider here only the sound made by Tibetan metal singing bowls, or how sodium chloride is described as tasting salty, rather than metallic (see also Ben Abu et al., 2018), despite the latter being a metal salt (see Table 1 for a review of the taste properties associated with various metal salts/minerals).

**Table 1.** Taste Qualities and Threshold Concentration of Mineral Salts in Different Mediums, With or Without Nose Clip (Reprinted From Delompré et al., 2019).

Formula	Name	Taste qualities	Threshold concentration (M)	Medium
NaCl	Sodium chloride	Salty	^a^8.0 × 10^−3^ (Fazzalari, 1973)	Water
KCI	Potassium chloride	Salty, bitter, metallic	^a^1.7 × 10^−2^ (Fazzalari, 1973)	Water
CaCl_2_	Calcium chloride	Bitter, salty	^a^1.0 × 10^−2^ (Fazzalari, 1973)	Water
MgCl_2_	Magnesium chloride	Bitter	^a^1.5 × 10^−2^ (Fazzalari, 1973)	Water
MgSO_4_	Magnesium sulphate	ND	^a^4.6 × 10^−3^ (Fazzalari, 1973)	Water
LiCl_2_	Lithium chloride	Salty, sour	^a^2.5 × 10^−2^ (Fazzalari, 1973)	Water
Nal	Sodium iodide	ND	^a^2.8 × 10^−2^ (Fazzalari, 1973)	Water
CuSO_4_	Copper sulphate	ND	^a^6.2 × 10^−6^ (Young et al., 1996)	Water
Na_2_SO_4_	Sodium sulphate	Salty, bitter	^a^1.7 × 10^−3^ (Lopez et al., 2017)	Water
CaSO_4_	Calcium sulphate	Salty, bitter	^a^8.3 × 10^−4^ (Lopez et al., 2017)	Water
Na_2_NO_3_	Sodium nitrate	Untasty	^a^1.6 × 10^−3^ (Lopez et al., 2017)	Water
CaNO_3_	Calcium nitrate	Untasty	^a^1.6 × 10^−3^ (Lopez et al., 2017)	Water
FeSO_4_	Ferrous sulphate	ND	^a^9.9 × 10^−5^ (Lim & Lawless, 2006)	Deionised water
FeSO_4_	Ferrous sulphate	Metallic	^a^3.0 × 10^−5^ (Epke & Lawless, 2007)	Deionised water
FeSO_4_	Ferrous sulphate	ND	1.6 × 10^−4^ (Epke & Lawless, 2007)	Deionised water
FeCl_2_	Ferrous chloride	ND	^a^6.6 × 10^−5^ (Lim & Lawless, 2006)	Deionised water
FeCl_2_	Ferrous chloride	ND	^a^6.4 × 10^−5^ (Epke & Lawless, 2007)	Deionised water
FeCl_2_	Ferrous chloride	ND	2.27 × 10^−4^ (Epke & Lawless, 2007)	Deionised water
CuSO_4_	Copper sulphate	Bitter, astringent, metallic	^a^7.8 × 10^−6^ (Epke & Lawless, 2007)	Deionised water
CuSO_4_	Copper sulphate	Bitter, astringent, metallic	2.46 × 10^−5^ (Epke & Lawless, 2007)	Deionised water
CuCl_2_	Copper chloride	ND	^a^8.2 × 10^−6^ (Epke & Lawless, 2007)	Deionised water
CuCl_2_	Copper chloride	ND	1.56 × 10^−5^ (Epke & Lawless, 2007)	Deionised water

*Note.* ND = not determined.

a Without nose clip.

At the same time, the sweet taste of lead has affected everyone from the ancient Romans, who famously used this highly malleable metal for their water pipes (e.g., Nriagu, 1983; Reddy & Braun, 2010; though see also Delile et al., 2014) through to Ludwig Van Beethoven. The latter’s deafness has been put down to his chronic consumption of wine tainted with lead (M. H. Stevens et al., 2013). During, Beethoven’s era, lead was added illegally to inexpensive wine to help improve the taste/flavour.^9^ Ancient Romans would also boil wine in lead to add sweetness, namely, lead acetate—a sweet salt of lead that was known as *sugar of lead* (Rhodes, 2012). Although, in this case, a bit like the sound of brassy instruments mentioned earlier, while literally tasting this poisonous metal (lead), the gustatory sensation is described as *sweet*, rather than *metallic* (in contrast to the salts of sodium and potassium while are primarily described as tasting salty).^10^

## Chemosensory Induction of Metallic Sensations

More generally, one might ask whether metallic chemosensations are anything more than just an off-flavour (or dysguesia; as once suggested by Frank et al., 1992; see also K. Yanagisawa et al., 1998), or whether instead they might constitute a biologically relevant sensing pathway (see Pirkwieser et al., 2021). Here, it is worth highlighting the importance of trace elements to human nutrition (Underwood, 2012). Nowadays, various staple foods, including everything from bread to breakfast cereal are fortified with iron, and other trace elements (e.g., especially for the benefit of pregnant mothers and their offspring). As Schiffman (2000, p. 158) notes:

The fact that metal salts induce taste sensations that are discriminable from one another makes sense from an evolutionary point of view. There are nutritional requirements in humans for metal salts including copper (1.0 to 2.6 mg/day), iron (15 mg/day), and zinc (15 mg/day) (Melchior & Jaber, 1996).

Schiffman (2000, p. 158) continues: “Thus, the ability to detect and discriminate among the tastes of these trace elements with metallic-like taste qualities has survival value.” There are also serious health consequences associated with the overconsumption of certain metals. Some Europeans (including members of the first author’s immediate family) have two copies of the faulty gene that results in haemochromatopsia,^11^ meaning that they find it difficult to process the iron in their diet, thus increasing the risk of various negative health outcomes (Pilling et al., 2019). As such, it would presumably be beneficial were we able to detect (i.e., to perceive) iron in food or drinking water (Hoehl et al., 2010; Mirlohi et al., 2011). That said, the ability to detect iron, rather than the more general metallic sensing, would be much more relevant for those suffering from this particular condition (cf. Schiffman, 2000, on the ability to distinguish between different metal salts; and Delompré et al., 2019, on nutrient tasting).^12^

There may be scope here to consider metallic taste judgements more as cognitive assessments rather than necessarily a straightforwardly perceptual response. There is certainly plenty of evidence to suggest that people’s affective response, both perceptual and neural, to certain olfactory stimuli (such as isovaleric acid) can diverge dramatically as a function of whether the odourant is labelled as *parmesan cheese* or *sweaty socks* (De Araujo et al., 2005; Djordjevic et al., 2008; Grabenhorst et al., 2008; Herz & von Clef, 2001). Hence, were patients, for example, told that the unusual mouth sensation they experienced was “metallic,” this might lead to a very different interpretation of the sensation than if some other descriptor was used instead. Relevant here, Lawless et al. (2005, p. 185) note that: “Metallic taste was reported from stimulation with ferrous sulfate solutions, from metals and from electrical stimuli. However, reports of metallic taste were more frequent when the word ‘metallic’ was presented embedded in a list of choices, as opposed to simple free-choice labelling.” Along similar lines, given the often-mentioned confusion that tasting panels often have with correctly labelling bitter and sour (e.g., Hettinger et al., 1999; O’Mahony et al., 1979), it might be interesting to assess whether the label that panellists give is linked to, or alters, their hedonic response.

### Sensory Science of Metallic Chemosensory/Oronasal

Working with a trained sensory (descriptive) panel, Epke et al. (2009) conducted a couple of studies in which they presented ferrous sulphate and copper sulphate to their participants either with, or without, nasal occlusion, and either with, or without, oral contact. The metallic taste and aftertaste (see also Yang & Lawless, 2005) that these stimuli gave rise to, was reduced both by nasal occlusion and by the prevention of oral contact (by holding the liquid in the mouth in a plastic lid). Given that, at the concentrations used, there was no orthonasal olfactory metallic smell, these results were taken to show that metal salts, especially ferrous sulphate generate lipid oxidation products in the oral cavity that are perceived retronasally as metallic flavours.

**Figure 1.**
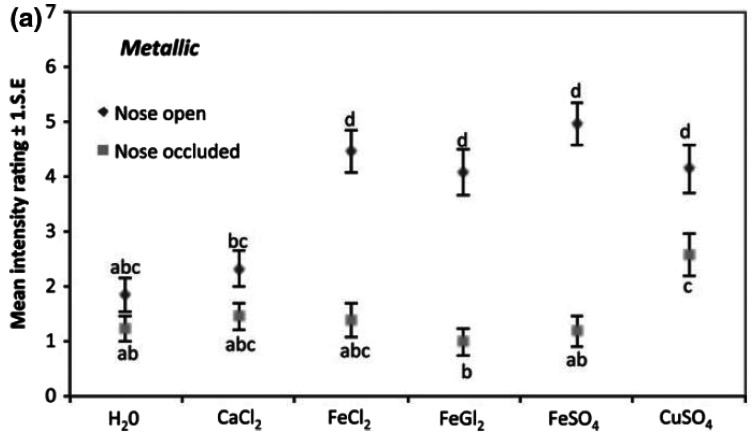
Mean metallic intensity ratings with the nose open and occluded for five divalent metal salts. Data points with different letters _abcd_ show significant differences (*p* < .05) across samples and nose conditions according to the Tukey post hoc test (reprinted from Skinner et al., 2017).

More recently, Skinner et al. (2017) conducted a carefully controlled study involving the tasting of various metal salts with the participants sometimes holding their nose closed in an attempt to identify any purely gustatory contributions to tasting. The researchers investigated the orthonasal contribution to metallic sensations. While headspace analysis of divalent salt solutions failed to reveal the presence of volatiles above the level of detection, some samples could be discriminated from water references when participants sniffed the headspace. The complex pattern of results (see Figure 1), was consistent with the existence of different chemosensory routes to experiencing metallic sensations. They not only demonstrated the origin of ferrous metallic sensation as retronasal smell but also the orthonasal capacity to detect ferrous salts. Metallic sensations evoked by copper could not be linked to either orthonasal or retronasal smell. Taste may thus perhaps be implicated in this case, though the involvement of trigeminal mechanisms cannot always be discounted (Skinner et al., 2017). The authors concluded that lipid oxidation products, formed after the sample volatiles came in contact with tissue in the nasal cavity, were perceived by sniffing. Such results are perhaps consistent with the suggested role of lipid oxidation, chelating agents, and antioxidants in the development of metallic flavour oral cavity (Ömür-Özbek et al., 2012).

The lipid oxidation story is intriguing because it raises the possibility that certain metallic sensations might only be experienced retronasally (but not orthonasally; Bodyfelt et al., 1988). However, this is unlikely to be the whole story regarding metallic chemosensation, given that when touched or pickled, iron is associated with distinct orthonasal aromas linked to (skin) carbonyl compounds and organophosphines (Glindemann et al., 2006). The latter researchers reported that metallic odourants were elicited in their study by iron-containing surfaces, either upon skin contact through the reaction of ferrous ions with skin peroxides, yielding aldehydes and ketones, or as a result of exposure to acid, producing volatile organophosphines mainly responsible for the “garlic carbide” metallic odour. Glindemann et al. (2006) were led to conclude that: “The typical ‘musty’ metallic odor of iron touching skin (epidermis) is caused by volatile carbonyl compounds (aldehydes, ketones) through the reaction of skin peroxides with ferrous ions (Fe^2+^) that are formed in the sweat-mediated corrosion of iron” (p. 7008). This they distinguish from: “The ‘garlic-carbide’ metallic odour of phosphrous- and carbon-rich cast iron and steel under attack by acid, dominated by volatile organophosphines.” Glindemann et al. discuss the mushroom-like metallic odour of 1-octen-3-one, which is, once again, the result of lipid oxidation. However, the smell (i.e., orthonasal olfaction) should really be classified as a human body odour rather than the smell of metal as such. Glindemann et al. (2006) conclude that:

Overall “skin–iron odor” mechanism: The metallic odor from iron contacting skin is surprisingly a type of human body odor. Sweaty skin corrodes iron metal to form reactive Fe^2+^ ions that are oxidized within seconds to Fe^3+^ ions while simultaneously reducing and decomposing existing skin lipidperoxides to odorous carbonyl hydrocarbons that are perceived as a metallic odor. This fast reaction creates the sensory illusion that it is the “metal in itself” that we smell right after touching it. Similar mechanisms underlie the skin–metal odors of iron, copper, and brass (Supporting Information) used as every-day objects. (p. 7007)

One of the challenges when working in the area of chemosensory metallic sensations is defining what exactly constitutes the semantic descriptor of “metallic.” As far as regular consumers are concerned, it would seem fair to say that the term is not clearly understood. Indeed, it has been suggested that its meaning may differ across individuals (Panovska et al., 2009), across samples (Schiffman, 2000), and may be influenced by whether assessors have been trained with a specific reference metallic sample prior to testing (see Lawless et al., 2004).^13^ This would make it a *polythetic* term or category, in contrast to most of the terms we use to order our experience, which tend to be *monothetic* terms—that is, terms which group things in classes by reference to common features: A polythetic class does not require that one feature be evinced by every instance, there can be an array of features only some of which overlap from instance to instance (Needham, 1975). Indeed, assessors have sometimes been found to report the metallic sensation elicited by different cations (metal salts) as a *different type of metallic* (Schiffman, 2000). As such, providing a single metallic reference in laboratory research may be too restrictive a practice, despite the fact that many consumers struggle to know what exactly the sensations that are described as metallic actually taste like (in a sense, this is perhaps a bit like the notion of *umami* for those with a western palette, or perhaps the mysterious concept of minerality among wine-tasters; Maltman, 2020).

It is also important to consider, as part of the cognitive assessment of metallic oronasal sensation, that the choice of descriptors seems to be dependent on previously learned associations. In a study that investigated the sensory properties of iron, calcium, zinc, and magnesium salts, the different metallic sensations were defined according to the panellists’ experiences with a variety of commercial products (Yang & Lawless, 2005). In another study, the participants described the metallic flavours from samples of ferrous and cupric salt solutions using terms such as *rusty metal*, *copper coin*, *bloody*, *amalgam dental filling,* *fishy,* and *tap water* (Hong, 2011).

In fact, it has been suggested that odour naming is nonconsensual as people tend to associate certain odours to different contexts or products in which they are found. It is common to describe an odour using descriptions of the odour source (e.g., flower odours described as *cleaning supply* or *bathroom freshener*; Hickman, in press; Jonsson & Stevenson, 2014; Sulmont-Rosse et al., 2005). Given the retronasal contribution to the perception of metallic flavour, it is expected that different types of metallic flavours will be associated and described according to that flavour’s probable source (see also Spence, 2020b, on the importance of source attribution to determining how people think about olfactory stimuli).

At the same time as considering the taste, aroma, and flavour of metals themselves, it is worth noting that the presence of certain metals, such as copper and silver, have been shown to facilitate the detection of other olfactory stimuli, such as thiols (Haag et al., 2020; Li et al., 2016). In a sense, this indirect action can perhaps be considered as akin to the taste-/flavour-enhancing properties of kokumi (Maruyama et al., 2012). The presence of zinc salts, meanwhile, has been shown to be a potent inhibitor of the basic tastes of sweetness and bitterness (>70% reduction in taste) while leaving salt, savoury, and sour tastes unaffected (Keast, 2003). See also Breslin and Beauchamp (1997) on taste-taste masking, and Davidson et al. (1999) on the way in which tastants can enhance aroma/flavour perception (e.g., in the case of menthol chewing gum; see Spence, 2021, for a review).

Metal ions activate the human taste receptor TAS2R7 (Behrens et al., 2019; Wang et al., 2019), hence suggesting a link to the perception of bitter tastes (Reith & Spence, 2020). Indeed, consistent with such a view, those reporting chemotherapy-induced metallic sensations have been reported to exhibit enhanced sensitivity to bitterness (IJpma et al., 2017). The T1R3 taste receptor has been proposed as a calcium taste receptor in humans (Tordoff et al., 2012). Meanwhile, artificial sweeteners and salts producing a metallic taste sensation have been reported to activate TRPV1 (Riera et al., 2007; see also Riera et al., 2009), hence suggesting a role for the trigeminal nerve in conveying at least some metallic sensations. In the oral mucosa, these receptors are expressed by those trigeminal nerve endings that respond to painful stimulation, temperature, acid, and a wide range of molecules including alcohol and vanilloids (e.g., including the capsaicin from chilli pepper; see Reith & Spence, 2020; Riera et al., 2007).

## Eating and Drinking With Metal

For millennia, metallic serviceware has primed notions of elegance, luxury, and premiumness (Carvalho & Spence, 2021; cf. Ariely, 2008, pp. 159–160). As the Roman gourmand Apicius suggested in the first-century AD in the book *Cookery and Dining in Imperial Rome*: “an expensive silver platter would enhance the appearance of this dish materially” when referring to the plating of his Apician Dish (number 141; Apicius, 1936, p. 103). Relevant here, a metallic appearance appears to set positively valenced expectations that can then carry over to influence the subsequent taste experience (Carvalho & Spence, 2021). Metallic cups have been shown to evoke positive judgements (i.e., elegance), which may, in turn, improve the coffee drinking experience (e.g., J. Liu et al., 2015; Thumfart et al., 2008; H. Yanagisawa & Yuki, 2011).

The appeal of serviceware made of, or plated with, valuable metals has been documented more recently too with Napoleon Bonaparte once sporting a platinum cutlery set (Aldersey-Williams, 2011). Crumpacker (2006, p. 190) describes a table service for 18 diners sold at Sotheby’s auction house in 1994. Apparently, each setting had 18 pieces including an ice-cream fork and spoon and an oyster spoon. Six of the pieces in each setting had been fashioned from solid gold. Meanwhile, while Charlie Chaplin was at the height of his fame, living in California, he would serve his guests meals from gold plates (Arce, 1979, p. 106). Tastevins have traditionally been made of silver, as, of course, are rings and many other forms of jewellery. Opaque pewter tankards were once popular drinking receptacles in Europe (Monson-Fitzjohn, 1927).

### On the Enduring Popularity of Metallic Tableware

People have been using metallic serviceware for centuries, including both metallic plateware and cutlery (often incorporating precious metals; e.g., Aldersey-Williams, 2011; Brown, 2001). For instance, according to Woolgar (2018, p. 19):

Most food that was destined for elite tables was produced with a limited range of serving dishes in mind of silver or silver gilt. The use of solid gold vessels and utensils was rare, although they might be used by monarchs. (Gold spoons associated with Richard II and English queens: Woolgar, 2006, p. 240; Stratford, 2012, pp. 147–148, 267–268.) The use of metals, with a shine, or themselves of virtuous composition, was something that added to the beneficial effects of what was about to be eaten. Shining food in shining dishes brought the maximum beneficence from light. Just as the evidence for cooking and colour relates almost exclusively to an elite level, so outside the level of the elite, or even in documents at this level, it is exceptional to have descriptions of dishes beyond those made of precious metals.

Indeed, ancient elites in most cultures preferred tableware to be made of precious metals. For instance, in the Bible, the Book of Esther presents a passage about a banquet lasting for 7 days at the king’s palace, in which metals such as gold and silver were described to be present not only in the tableware but also displayed in the dining environment:

There were white cotton curtains and blue hangings caught up with cords of fine linen and purple to silver rings and marble pillars, and also couches of gold and silver on a mosaic pavement of porphyry, marble, mother-of-pearl and precious stones. Drinks were served in golden goblets, goblets of different kinds, and the royal wine was lavished according to the bounty of the king. (Old Testament, The King’s Banquets, Esther 1:5)

### Metallic Plateware

While the use of precious metals and precious metal inlays in plateware can all too easily appear garish in the contemporary, often brightly illuminated, indoor environment (at least to the Western eye), it is worth remembering that the glitter of gold inlay in Japanese lacquerware would have glinted in a much more appealing manner in centuries gone by. That is, when houses were illuminated by candlelight or gas lamps, prior to the advent of bright artificial modern illumination (Tanizaki, 2001). The Nobel laureate, writer Junichirō Tanizaki (2001), in his extended essay on aesthetics *In Praise of Shadows*, draws attention to the close interplay that exists, or better said, once existed, between architectural design and food/plateware design in traditional Japanese culture. For instance, Tanizaki talks of how black lacquerware, decorated perhaps with flecks of silver and gold would glisten in the darkness under candlelight: “Their extravagant use of gold, too, I should imagine, came of understanding how it gleams forth from out of the darkness and reflects the lamplight” (p. 23). Tanizaki continues:

Lacquerware decorated in gold is not something to be seen in a brilliant light, to be taken in at a single glance; it should be left in the dark, a part here and a part there picked up by a faint light. (p. 24)

**Figure 2.**
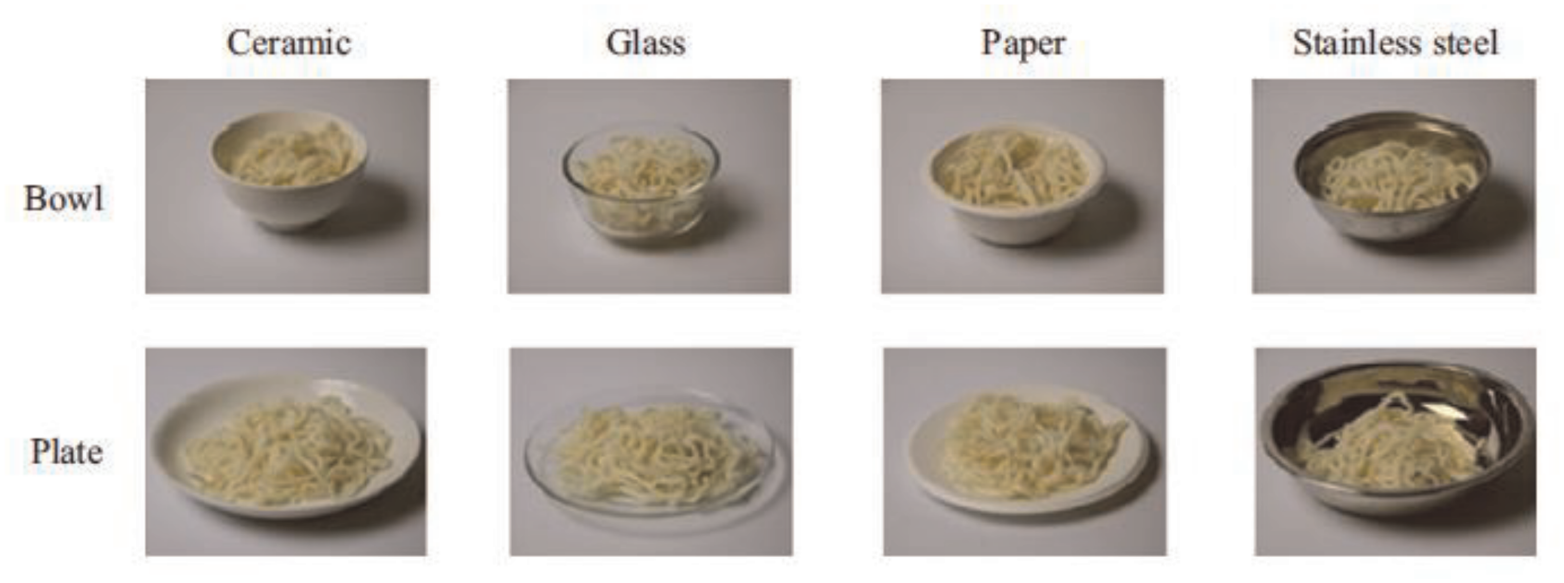
Images of noodles presented in bowls and plates of different materials. When the noodles were presented in the metallic-looking stainless-steel bowl or plate, ratings of the noodles declined among the western participants who took part in this cross-cultural study (reprinted from Zhou et al., 2015).

More recently, researchers have started to investigate the influence of metallic plateware on people’s perception of the food served thereon. So, for example, the participants in an online study conducted in mainland China by Zhou et al. (2015) had to rate noodles (natural in appearance, or else coloured red, green, or yellow) displayed on/in ceramic, glass, paper, or stainless steel plates or bowls (i.e., there were eight presentations in total; see Figure 2).^14^ The participants reported which basic taste term came to mind first and their feelings about the noodles (including ratings of familiarity, pleasantness, and naturalness). However, the results revealed that the material of the plateware exerted only a very modest impact on the pattern of results obtained, and there was no evidence that the stainless steel plates/bowls elevated the participants’ subjective ratings of the noodles. Such results might be taken to suggest that the ubiquitous use of stainless steel in contemporary society means that it no longer has any particularly positive associations. Given Tanizaki’s (2001) intriguing suggestions, mentioned in the preceding paragraph, it would perhaps be interesting to repeat Zhou et al.’s experimental design with Japanese lacquerware plates under a variety of lighting conditions (cf. van der Heijden et al., 2021).

### Metallic Cutlery

Throughout recent history, cutlery has been manufactured out of various materials. Historically, wood, bone, and ceramic spoons were commonly used because of the fact that the only metals that were affordable at the time were iron, brass, bronze, and pewter.^15^ According to Himsworth (1953, p. ix), stainless steel was introduced as a suitable metal for cutlery in 1914. Its subsequent success resulted in a radical reduction in the range of materials used (see Spence & Piqueras-Fiszman, 2014), so that nowadays, stainless steel is by far the most commonly used material for cutlery in the west (along with silver).^16^ At the same time, however, stainless steel’s very ubiquity has resulted in it losing any symbolic meaning that it may once have had, on first being introduced, and hence there is little evidence of sensation transference (see Chen et al., 2018; Zhou et al., 2015).

Researchers have had people rate the taste attributes associated with licking spoons, in the absence of any food stimulus (P. Howes et al., 2014; Laughlin et al., 2009, 2011). For instance, Laughlin et al. (2011) investigated the sensory-discriminative effects of metallic sensation arising from spoons that had been coated with a range of different metals. The results revealed that gold, silver, zinc, copper, tin, chrome, and stainless steel plated spoons yielded different taste sensations among the participants. The researchers investigated the sensory-discriminative and hedonic effects of metallic tastes arising from seven spoons plated with different metals: gold, silver, zinc, copper, tin, chrome, and stainless steel. While blindfolded, participants evaluated the taste of each of the spoons and rated the following attributes on a 7-point scale: cool, hard, salty, bitter, metallic, strong, sweet, and unpleasant. Laughlin et al.’s (2011) results demonstrated that the gold, chrome, tin, silver, and stainless steel spoons did not differ significantly for any of the rated attributes, but they were significantly different from the zinc and copper spoons. Gold and chrome were rated as the least metallic, least bitter, and least strong tasting of the spoons, whereas zinc and copper spoons had the strongest, most metallic, most bitter, and least sweet taste.

**Figure 3.**
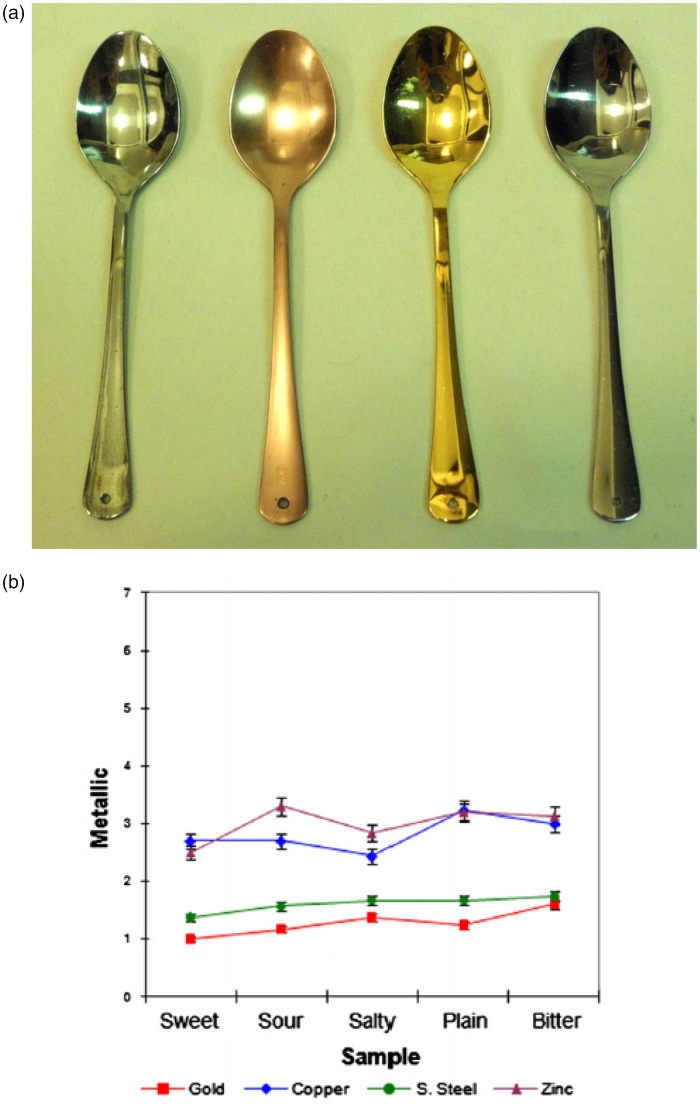
A: The four metal-plated spoons (zinc, copper, gold, and stainless steel) used by the blindfolded participants in a study by Piqueras-Fiszman et al. (2012). B: The summary results highlighting the impact of the different spoons on ratings of metallic sensory attributes of cream samples (plain, or adulterated with one of the four basic tastes; reprinted from Piqueras-Fiszman et al., 2012).

Metals have a taste, and what is more metallic cutlery has been shown to influence the taste of the foods that are sampled from them (e.g., Piqueras-Fiszman et al., 2012). Piqueras-Fiszman et al. had their participants (*N* = 30) taste and rate cream samples having different tastes (sweet, sour, bitter, salty, and plain) from each of four teaspoons that had been plated with different metals (gold, silver, stainless steel, and zinc; see Figure 3A). Plating ensured that all of the spoons were exactly the same size, shape, and weight. The participants were blindfolded while tasting the creams so that the visual appearance of the spoons did not bias their expectations regarding taste. As such, these researchers were able to isolate the gustatory contribution to the multisensory tasting experience. The results revealed that, in addition to transferring a somewhat metallic (see Figure 3B) and bitter taste, the zinc and copper spoons also enhanced to a greater or lesser extent, each cream’s dominant taste. Intriguingly, however, the metallic taste of the copper and zinc spoons did not appear to deleteriously affect the participants’ ratings of the pleasantness of the samples. When considering the taste qualities imparted by different metals, one might consider the possible role of their differing ionisation tendencies. However, the authors are not aware of any research connecting these phenomena.

**Figure 4.**
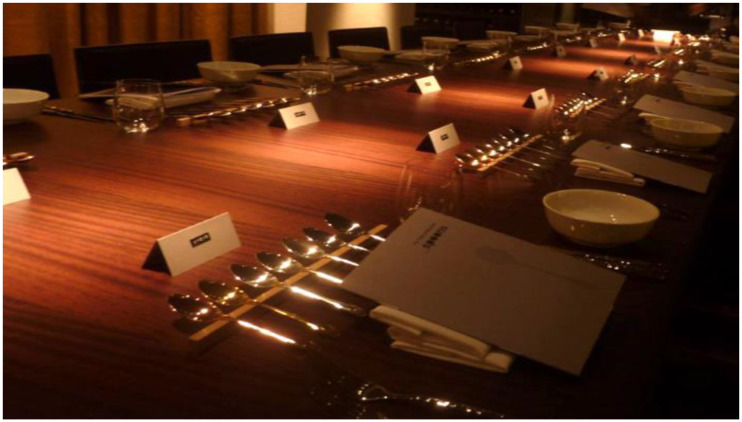
The metal-plated spoons that diners had to taste the meal served at Quilon restaurant in London in 2012 (reprinted from Spence & Piqueras-Fiszman, 2014).

The publication of Piqueras-Fiszman et al.’s (2012) results then led on to a multicourse tasting dinner that was held in the Quilon restaurant in central London (http://www.quilon.co.uk/) where the guests (including the first author) were given spoons made of seven different metals to taste with (see Figure 4). However, the powerful flavours of the Indian cuisine that were served on the occasion tended to overpower the gustatory impact of the metals of the spoons, at least for the first author (see also Dunlop, 2012).

### Metallic Drinking Receptacles

**Figure 5.**
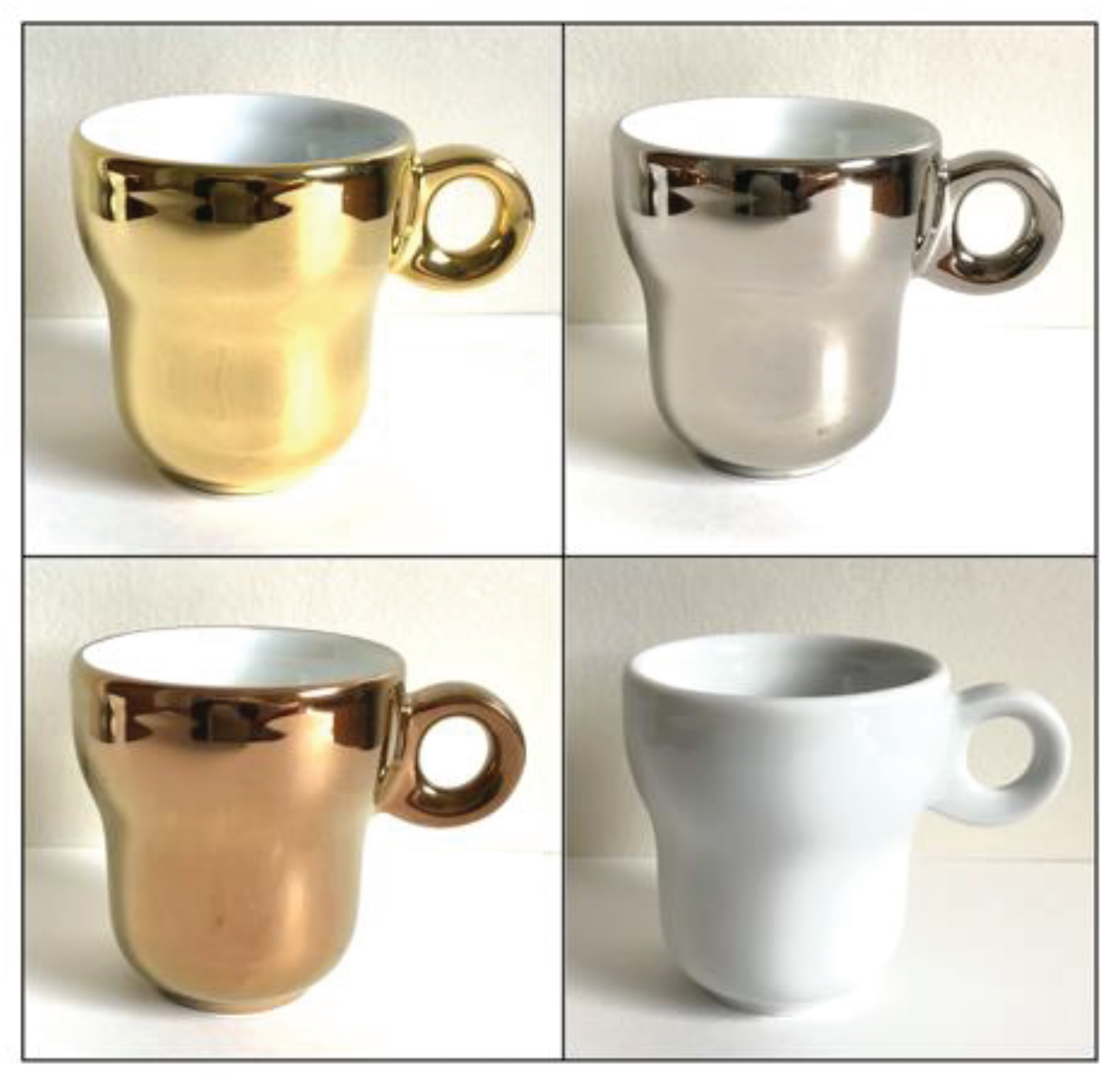
The various metallic-coated coffee cups that were presented to participants in Carvalho and Spence’s (2021) recent study. From top-left to bottom right: gold, platinum, brass, and uncoated (reprinted from Carvalho & Spence, 2021).

Carvalho and Spence (2021) conducted a study in which their Brazilian participants tasted and rated two coffees (Brazilian and Kenyan) with distinctive sensory profiles served from one of four white ceramic cups whose outer surface had been covered with gold, platinum, bronze, or else was left uncoated (see Figure 5).^17^ In total, 51 coffee experts and 132 regular coffee consumers took part in the study. The results revealed that higher hedonic and sensory ratings were given to the Brazilian coffee when tasted from a bronze cup and to the Kenyan coffee when tasted from the gold cup. The bronze cup enhanced sweetness ratings for both coffees among the experts, while the bronze and the gold cups increased the ratings of aroma, elegance, and liking for the Brazilian and Kenyan coffees, respectively. The platinum-coated coffee cup significantly increased ratings of the metallic note in the taste of both coffees in both groups of tasters relative to the other three cups.^18^ However, this did not lead to any obvious reduction in liking relative to the coffees sampled from the other cups. Note, though, that in contrast to the studies with spoons that were just mentioned, the metallic outer surface of the cups in Carvalho and Spence’s study is only likely to have made contact with the tasters’ lower lip, and not with any of the taste buds directly, nor with the drink itself. As such, the impact of the metallic outer appearance of the cups is likely to have exerted its effect solely as a result of any associations that may have been primed visually. In future research, it would be interesting to assess people’s pretasting expectations on viewing the metallic-coated cups as well as their subsequent experience on tasting the coffees (cf. Carvalho & Spence, 2019; Spence, 2018). Starbucks has been investing in reusable mugs (and straws) made of stainless steel available in a variety of outer metallic coatings such as bronze and copper (see https://www.starbucks.com.hk/coffeehouse/merchandise/starbucks-heritage-series).

### Metallic Packaging

Silver foil has long been popular as an inner packaging material (as in the case of Nestlé’s KitKat confectionary; Hine, 1995). Gold foil also makes an occasional (distinctive and eye-catching) appearance in the covering of the Lindt Goldhaser Easter chocolate bunny (Gangjee, 2018, p. 84). That said, the perception of metallic packaging is no longer quite so positive these days, given growing concerns about sustainability, and so on (Steenis et al., 2017; cf. Velasco & Spence, 2019). Indeed, Nestlé switched the iconic foil inner sleeve (and paper outer) for their KitKat after 65 years for a single flow-wrap plastic foil packaging in 2001 (see Anon, 2001; Bashford, 2001). Commentators have since been divided over whether this was a good idea or not (in part given the ritual that had developed among consumers around running a finger over the line in the foil seal (see Spence, in press). When used in outer packaging, a silver appearance tends to be associated with the packaging of dairy products in the United States while being associated with fresh seafood in Norway; Burton, 2008; Heide & Olsen, 2017). Meanwhile, gold is not widely associated among customers with a particular product category or brand (Labrecque & Milne, 2013), excepting perhaps Nescafe’s Gold Blend instant coffee, Starbucks Gold Label, and the Lindt gold bunny mentioned a moment ago. As such, this visual appearance property can effectively be used to convey luxury/elegance (Sperdea & Criveanu, 2014), as has been successfully achieved in the world of (heraldic-looking) wine labels (Heine et al., 2016; Pelet et al., 2020). What is more, many adverts for luxury items including alcoholic beverages use extrinsic attributes such as gold and shininess in order to convey prestige and style (Kirmani & Zeithaml, 1993). The same outcome has, on occasion, also been achieved by the use of metallic menus (Magnini & Kim, 2016).

### Sensation Transference From Metallic Serviceware, and the Symbolic Associations With Metals

Precious metals tend to be symbolically meaningful (Chadwick, 2021). However, the symbolism attached has, in some cases at least, changed over the decades/centuries. According to Chris Woolgar (2018, p. 18), in the Medieval era, a certain Master Chiquart recommended that gold coins be put into the dishes prepared for the sick (see Scully, 2010). This usage was consistent with the chromotherapy of the epoch. Gold shavings were added to medicinal preparations on account of their association with the power of the sun (just as emeralds were valued for their “life-giving” greenness and therefore worn next to the skin). “In his *Canterbury Tales* Chaucer jokingly related of the physician in the party of pilgrims that he loved gold—for its medicinal properties” (D. Howes & Classen, 2014, p. 41). Meanwhile, the Italian Futurists were once so enamoured with aluminium foil (as a Futuristic material that had just been invented) that they would plaster their dining rooms with it (see Berghaus, 2001; Spence, 2017). Elsewhere, in *The Futurist Cookbook* (Marinetti, 1932/2014), one also finds a recipe for roast chicken filled with metallic ball-bearings (again more for their symbolic meaning than for their ability to enhance the taste/flavour of the dish).

One might consider whether this reflects an example of hedonic “sensation transference” (Cheskin, 1957, 1972). Cheskin himself was mostly interested in the transfer of (our feeling about the) sensory attributes from product packaging to the perception of the contents. However, the same principle could equally well be applied to the observed transfer of a person’s expectations and feelings concerning relevant product-extrinsic cues (e.g., metallic coating) to their evaluation of the intrinsic property of the product (e.g., its quality). The term “affective ventriloquism” has been coined to refer to those situations in which sensation transference influences hedonic ratings of products (Spence & Gallace, 2011). Sensation transference is presumably more likely to occur with symbolically meaningful and valuable stimuli (such as precious metals).

The phenomenon of sensation transference is, however, especially interesting in the case of metallic, given the bivalent associations that have been repeatedly documented throughout this review, depending on the modality with which those sensations are attached. Here, one might want to ask (or know) whether the sensations that are transferred are those that are linked to the source stimulus modality (i.e., to metallic visual appearance, and so positively valenced; as in the case of Carvalho & Spence,’s, 2021, metallic coffee cup study) or to the target modality (namely, taste/flavour, in which case they might be negatively valenced). This would seem to be quite different from what happens in the case of crossmodal priming, where the associations tend to be attached to the target modality (e.g., Chen & Spence, 2011; B. Liu et al., 2012; Vallet et al., 2010).^19^

Furthermore, it is worth remembering that the sight of the cutlery made from precious (or unusual) metals may turn out to have a more pronounced influence on the tasting experience (Laughlin et al., 2011, e.g., as a result of sensation transference; Cheskin, 1957, 1972) than any direct effects attributable to the taste that metallic serviceware may sometimes impart. That said, tasting the plain cream from gold and stainless steel spoons in Piqueras-Fiszman et al.’s (2012) study gave rise to very similar ratings regardless of whether the participants had their eyes open or closed. At the same time, however, metallic appearance properties do not necessarily always connote *quality* or elegance (Piqueras-Fiszman & Spence, 2011). As has been mentioned already, certain metals, like stainless steel have become so ubiquitous that they may no longer be associated with elegance/luxury in quite the way that they once were (cf. Zhou et al., 2015). The symbolic meanings of precious metals can also vary as a function of culture (and over the course of history), as we have seen.

## Eating Metal/Metal Flavours

Silver hundreds and thousands provide a decorative topping to cakes and pastries, though here the intention is presumably to add some sparkly eye-appeal (cf. Giedion, 1948/1975; Kirchner et al., 2015) rather than necessarily to convey the illusion of eating something expensive (like a precious metal). By contrast, edible gold leaf has occasionally been incorporated into various ultra-expensive dishes, such as the gold flakes incorporated in one of the outrageously expensive dishes described by Spence and Piqueras-Fiszman (2014; Wharton, 2008; cf. Veblen, 1899/1992).^20^ Given that one of the only liquid metals (at or around room temperature) mercury, is highly toxic, and given the desirable nature of this material, one might consider whether augmented reality or virtual reality could be used to give drinks a metallic appearance (Ueda et al., 2020; cf. Spence, 2019). What would it be like to drink a truly golden liquid, rather than one that is merely described as such (such as is sometimes the case for Sauternes wine or urine)?^21^

A few years ago, the world-famous mixologist Tony Conigliaro added iron oxide to one of his Halloween-themed cocktails in the form of what looked like a few drops of blood (Spence, 2020a). The gustatory, or better said, chemosensory suggestion that guests were really drinking blood was key (rather than any metallic association with premiumness). Meanwhile, iron ore has also made an appearance in the dishes served by the idiosyncratic Swiss chef Stefan Weisner. For instance, at a 2019 conference on *The taste of art* held at Museum Tinguely in Basel, the assembled audience were served an iron-ore ice-cream dessert by the name of *Flint*, incorporating iron-ore distillate, iron-ore crumble, iron-ore chips, and was sprinkled with iron-ore salt (Spence, 2020a; Weisner & Müller-Alsbach, 2020). The fundamental aim behind such a presentation was not altogether clear to the first author. Intriguingly, the old wives’ tale suggesting that studding apples with iron nails and letting them marinate would help to keep anaemia away has been supported, in the animal (rat) model (see Anon, 1979).

### Why Is a Metallic Taste/Aroma/Flavour Always Unappealing?

Here one is left with the challenge of whether metallic could be transformed into a desirable (i.e., positively valenced) descriptor of chemosensory stimulation. After all, many other foul smelling/tasting foods such as ripe French cheese (such as Époisses de Bourgogne), or an unpleasant bitter-tasting food (such as coffee or beer), often come to be liked as a result of associative learning (e.g., Rozin & Zellner, 1985; Zellner et al., 1983) and mere exposure (e.g., Balogh & Porter, 1986; Pliner, 1982), so why not metallic taste/flavour sensations too?

In other words, while many people have wanted to eat precious metals, and occasionally to dine with metallic serviceware, metallic taste/flavour is typically a sensation that consumers choose to avoid. At the same time, however, one might consider whether metallic-smelling olfactory sensations might be unpleasant because they constitute one of the evolutionarily important components in blood (e.g., Nilsson et al., 2014; cf. Dunkel et al., 2014), and so act as an evolutionarily important warning signal. According to Dunn and Sanchez (2021), raw chicken also imparts something of a metallic taste.^22^ The iron contained in haemoglobin gives rise to a metallic odour on the oral mucosa or in the blood itself (Glindemann et al., 2006), with blood iron decomposing blood lipidperoxides. Meanwhile, Arshamian et al. (2017) have demonstrated that the volatile chemical *trans*-4,5-epoxy-(E)-2-decenal (E2D) found in mammalian blood tends to induce postural avoidance, increases physiological arousal, and enhances visual perception of affective stimuli in humans. E2D is generated by lipid peroxidation (Buettner & Schieberle, 2001), and, according to one of the authors of the study, E2D has a weak metallic odour, with a hint of generic *chemical odour*, while others have described it as having a smell that reminds them of an *untreated iron bar.* The suggestion is that the presence of this odourant may serve as an alarm signal across species. An important prey-associated odour stimulus for predators found the odourant organic “aldehyde” compound E2D, one of the volatile compounds in mammalian blood, In humans, this has a typical “metallic, blood-like” odour quality (Nilsson et al., 2014). The metallic smell of blood is likely going to be attractive to predators, such as wild dogs and Siberian tigers (Nilsson et al., 2014). Although, in contrast to Glindemann et al.’s suggestion that the smell of blood ought to be attractive to humans,^23^ it actually appears not to be, at least not according to Arshamian et al.’s (2017) findings.^24^

At the same time, of course, our theorising in this area is somewhat limited by the fact that despite all the research that has been conducted on the chemosensory induction of metallic sensations to date, we are still some way short of a full understanding of the underlying neural mechanisms. This, at least, was the conclusion of the latest review of metallic off-flavours by Pirkwieser et al. (2021) who note that:

The causes of this unpleasant perception are diverse, ranging from unfavorable concentrations of micronutrients, the use of artificial sweeteners, processing, packaging, and storage, to side effects of pharmaceutical or chemotherapeutic agents. However, the mechanisms behind metallic sensing and its contributions to taste, smell, and trigeminal nerve sensations are still poorly understood. (p. A)

## Should Metallic Be Considered as an Amodal, or ‘Ambimodal’ Quality?

How, then, should we describe metallic sensation? We have seen that a metallic sensation can be perceived by more than one sense, unlike colour or sound. Colour and sound are unimodal. It may therefore be tempting to consider metallic to be an *amodal* stimulus properties. However, we have also seen that a metallic sensation is evaluated differently depending on whether the stimulus is visual (in which case the sensation is typically described as pleasant) or chemosensory (in which case it is typically described as unpleasant, and unfamiliar, and *off* or tainted). How is this ambivalence to be explained? The concept of amodality cannot really help us here because it is too general to account for the bivalence (modality specificity) to the affective evaluation of the sensation. Nor can it explain the phenomenon of *sensation transference*, or crossmodal interaction, as, according to the concept of amodality, there are no modalities to be crossed. We propose that this conundrum can be resolved by introducing a new concept, the concept of the *ambimodal*, which we would like to suggest can be considered as occupying a position in-between the unimodal and the amodal. A brief excursion into the history of Western perceptual psychology will help clarify the roots of this neologism.

According to Aristotle’s perceptual psychology, there are *proper sensibles* which are perceptible by one sense (e.g., colour is the proper sensible of vision, sound of audition, smell of olfaction, etc.) and *common sensibles*, which can be perceived by more than one sense (e.g., figure, which is perceptible by both sight and touch; so too with the other common sensibles, such as extension, rest, and motion). Is metallic, then, a common sensible? If so, it would be unlike any of the others, so no. Aristotle would have needed to discriminate a sixth, metallic sense in the same way that umami has come to be identified as a fifth taste. But we know his views on that “there is no sixth sense in addition to the five enumerated—sight, hearing, smell, taste, touch” (*De Sensu* bk 3, ch 1, para 1; Aristotle, n.d.). Aristotle also proffered the idea of “the common sense” (*sensus communis*), though, which was basically a multimodal sensual faculty (see Heller-Roazen, 2007, p. 35). It could perhaps have accommodated the multiplicity of metallic sensations that have been reported.

The 17th-century British philosopher John Locke’s *Essay Concerning Human Understanding* is commonly seen as continuing in the Aristotelian tradition. But it actually introduced a rupture. According to Locke’s perceptual psychology (Locke, 2011), there are *primary qualities* and *secondary qualities.* The former took over from Aristotle’s common sensibles and the latter from his proper sensibles. However, the switch in order is significant, and so is the difference Locke imputed to their ontological status. Everything was sensible in Aristotle’s universe: for example, the universe was understood to be composed of four Elements (Air, Earth, Fire, and Water) which were, in turn, distinguished by reference to their differential combination of the four equally elementary tactile properties: hot, cold, wet, and dry (Earth was cold and dry, Fire was hot and dry, etc.). What is more, each sense was associated with a different Element: “Water [was] the element of sight (because the eye contains water), air the element of hearing, fire the element of smell and earth the element both of touch and of taste, which is a mode of touch” (Connor. 2015, p. 180). Not so for Locke. Influenced by the chemist Robert Boyle’s “corpuscular philosophy” (or atomistic conception of matter) and by his own experience examining life through the lens of a microscope, Locke suggested that the primary qualities were off the scale of human vision, hence infrasensible. He writes:

Blood to the naked eye appears all red; but by a good microscope, wherein its lesser parts appear, shows only some few globules of red, swimming in a pellucid liquor … that which is now the yellow colour of gold, would [if examined through a microscope] then disappear, and instead of it we should see an admirable texture of parts of a certain size and figure. (*Essay* vol 1, part 1, para 11)

To elaborate, primary qualities inhere to an object and are independent of any observer, hence can be considered as fundamentally amodal. Secondary qualities, by contrast, are properties that produce (unimodal) sensations in an observer such as the way blood appears red, but they are “a quality of the object” rather than “a power in the object”—that is, secondary qualities are an effect of the “various arrangements” (at the corpuscular or atomic level) of solidity, figure, spatial extension, rest, and motion.

There is an incipient sense in the writings of Boyle and Locke that the primary qualities are more “real” than the secondary qualities they generate in the mind of an observer. Boyle’s corpuscular philosophy would, in fact, go on to be substantiated by the Russian chemist Dmitri Mendeleev in 1869. The latter delimited the 63 elements of the Periodic Table of Elements. This discovery was world-shattering. In effect, Mendeleev dissolved the four Elements of the Aristotelian worldview (Earth, Air, Fire, and Water) into the 63 (now 118) elements of his Periodic Table, which are distinguished on the basis of their atomic number and recurring chemical properties alone. The magnitude of this redefinition of reality is astounding. Constance Classen (1998) discusses how the modern scientific philosophy transformed the cosmos from a “vibrant universe of sense” (as in Antiquity and throughout the Medieval period) into “what Alfred North Whitehead has called ‘a dull affair, soundless, scentless, colourless; merely the hurrying of material, endlessly, meaninglessly’” (p. 5).

Primary qualities are ontologically prior and fundamentally amodal, while secondary qualities are subjective and unimodal, according to the schema which emerges from the preceding account. Where does the metallic sensation fit within this schema? It does not, really, which is where the concept of the ambimodal comes in. The latter is an interstitial concept, or intercategory of sensation, neither unimodal nor amodal. The hybridity of this concept helps to explain numerous aspects of the metallic sensation. It is more than unimodal but less than amodal, for it is proper and common (in Aristotle’s terminology) at once. As an intercategory, it is inherently unstable, which explains why it is not very useful as a descriptor for purposes of laboratory research, the reason being that it denotes a *polythetic class* (Needham, 1975). As an intercategory, it is generative of ambivalence, which explains the bivalence to its evaluation across modalities (visual vs. chemosensory). As an intercategory it is something of an abomination: that is, it fits the anthropologist Mary Douglas’ analysis of the concepts of purity and pollution in *Purity and Danger*: according to Douglas, abominations are “matter out of place” due to the way they straddle boundaries or mix that which should be kept separate according to a given classificatory schema (Douglas, 1966). This helps explain why, in the case of chemosensory perception, the metallic sensation is perceived as “off” (i.e., impure, unfamiliar) instead of spot on (like colour). The lingering quality of the metallic sensation would also render it more *out of place* than *in*. The difficulty in categorising it as a basic taste, an olfactory sensation, or a flavour is further testimony to its ambipotentiality. Glindeman et al.’s (2006) observation that people are *perplexed* by the metal odour given off by iron metal objects when touched may be attributable to the skin-iron conjunction being perceived as polluting. Finally, it may be surmised that the reason metalsmiths are perceived as *dirty* (van Beek, 1992) stems from their close association with this highly multifarious and mutable sensation.

It is interesting to speculate on how differently Aristotle’s perceptual psychology would have turned out had he commenced his analysis with the ambimodal sensation of the metallic rather than the unimodal sensation of colour, sound, or scent, but that is the subject for another paper. In that future paper, in addition to elaborating further on the implication of this concept of the ambimodal, we plan to examine how the categories of Peircean semiotics can help enucleate the conundrum of the metallic sensation.

## Conclusions

In conclusion, *metallic* constitutes one of the most intriguing of all material properties (see Komatsu & Goda, 2018; Spence, 2020c, for reviews), both because of its differing connotations/semantic associations in different cultures and at different points in history (after all, it is hard to imagine anyone nowadays getting as excited as the Italian Futurists once were by tin foil; cf. Berghaus, 2001; Marinetti, 1932/2014; though see also Kingdon, 2018), but also because of the very different valence associated with sensations that people choose to describe as metallic, depending on the modality with which they happen to be attached. The term *metallic* often seems to cover a group of distinct sensations rather than just a single one, regardless of the sense involved (see Schiffman, 2000), it represents a *polythetic class* in other words (Needham, 1975). Visual metallic sensations are typically positively valenced, whereas those sensations that people choose to describe as metallic and happen to be associated with the stimulation of the chemical senses, typically negative (Reith & Spence, 2020). The concept of the ambimodal was introduced in an effort to more accurately evoke the ever shifting, multimodal aspects of this sensation. What has also emerged from this review is how when the term is used outside of the visual modality, the description of a sensation as *metallic* typically seems to imply that it is unfamiliar and unpleasant (see Meyer, 2013; New York Hall of Science, 2016; Nieukirk et al., 2016).^25^ As such, one might have expected that people would not like to eat with metallic cutlery, given the negative-valenced association. As we have seen, there is also an intriguing literature on sensation transference attached to this often highly symbolically meaningful stimulus attribute/material quality. However, sensation transference of positive attributes/valence associated with visual appearance (and the feel of weight; Cheskin, 1957, 1972; Michel et al., 2015) seemingly wins out over the crossmodal priming of the associated taste attribute (which is normally negatively-valenced).

Finally, the enduring question is raised as to why those chemosensory stimuli that happen to be described as smelling or tasting metallic should always be negatively valenced, given that many other food stimuli that are initially offensive come to be liked. One tentative suggestion here is that at least in certain cases there may be an evolutionarily preserved avoidance response linked to blood (Nilsson et al., 2014; though see also Glindemann et al., 2006, for an alternative view).

## Supplemental Material

sj-pdf-1-ipe-10.1177_20416695211037710 - Supplemental material for Metallic: A Bivalent Ambimodal Material Property? Click here for additional data file. Supplemental material, sj-pdf-1-ipe-10.1177_20416695211037710 for Metallic: A Bivalent Ambimodal Material Property? by Charles Spence, Fabiana M. Carvalho and David Howes in i-Perception

## Notes

1 Using this term helps us to sidestep the problematic associations that the term “amodal” has acquired (see Spence et al., 2013).

2 Intriguingly, however, the eye-appeal of shiny materials may be a phenomenon that is uniquely human (Silvia et al., 2018), given research suggesting that, contrary to European folklore, magpies do not appear to be attracted to shiny metallic stimuli (Shephard et al., 2015). According to one popular suggestion, our liking for glossy surfaces stems from our innate need for water (Meert et al., 2014). Such an explanation would, though, also seem to predict that glossy surfaces should be liked by other species that need water too.

3 For musicians, however, a “metallic” tone indicates that the string of the instrument is engaging in Helmholtz motion. This critical aspect of the vibrating string is needed to produce the highest quality of tone from an instrument (p. 149).

4 And one should perhaps also mention “heavy metal” as the descriptor of a style of music (Baker, 2013).

5 As Komatsu and Goda (2018) note: “Every sensory modality is involved in material perception. Not only that, material perception also has crossmodal aspects. For example, when we see a sweater made of fine wool, we can perceive that it will be soft and warm, or we can sense that a metal cup will be cold and hard to the touch” (p. 330).

6 As British Airways found to their cost when the titanium cutlery that had been specially designed for use on Concorde was rejected for feeling too light (see Spence & Piqueras-Fiszman, 2014). As Aldersey-Williams (2011) put it a decade ago, “We know the lighter aluminium spoon is easier to use, yet we prefer the silver because it ‘gratifies our taste’” (p. 257).

7 According to the summary statement accompanying Stark and Forss’s (1962) paper, “The ‘metallic compound’ previously reported as an important cause of off-flavour in oxidised dairy products has been identified as oct-l-en-3-one. The compound was found to have a flavour threshold value of one part in 10^9^ in butterfat and one part in 10^10^ in water. This compound was also isolated from oxidised safflower oil.”

8 Note here that the oral referral of retronasal olfaction to the oral cavity has also been suggested as a possible mechanism underpinning metallic sensations in the case of ferrous sulphate (Lawless, Schlake, et al., 2003).

9 The various illnesses suffered by another of the era’s great composers, George Frederic Handel, have also been put down to the overconsumption of cheap lead-tainted wine (Hunter, 2006).

10 There is clearly a chemistry angle here in terms of the interaction between foods and serviceware, as highlighted by discussion of why egg whites should be whipped in a copper bowl that appeared in *Nature* some years ago (McGee et al., 1984).

11 The HFE p.C282Y homozygous mutation is the main cause of the iron overload disorder hereditary haemochromatosis (type 1) in populations of European descent.

12 However, much like the Quilon meal with the seven tasting spoons that is described a little later, it is likely that although metal sensations are discriminable when presented in isolation, they will likely be masked/overpowered in any realistic food context.

13 A reference sample can be useful to establish a standardised perception of a sensation across different assessors. However, providing such a reference can also limit the conceptualisation of what constitutes “metallic” to a narrower criteria than is truly representative of different samples.

14 Note that meals often served from steel plates and bowls in public dining halls in China (Xie et al., 2009).

15 Although, according to Miodownik (2008), the latter would often add an unpleasant flavour to food. It has also been suggested (on a variety of online forums) that very high carbon steel knifes, such as Japanese Gyuto, may impart a metallic, or “iron” taste/flavour to some acidic foods, such as, for example, fruits.

16 Chopsticks, interestingly, still come in a range of materials depending on the country where they are used (see Spence & Piqueras-Fiszman, 2014).

17 Several companies offer porcelain coffee cups/mugs coated with metals such as gold, platinum, silver, bronze, and copper, for instance, Walkure Porzellan, Germany (walkuere-porzellan.com) and Wolff, Brazil (wolffbrasil.com.br).

18 Here, it is worth drawing the reader’s attention to the fact that metallic sensations are sometimes associated with certain kinds of coffee (Bicho et al., 2012).

19 Note that some intriguing questions also emerge here concerning the crossmodal semantic priming that might be elicited by the word “metallic” given its bivalent associations depending on the modality to which it is linked.

20 In Medieval England, golds and silvers in food were achieved by means of the use of thin sheets of foil of those metals in their dishes (Woolgar, 2018, p. 16).

21 Notice also how describing McDonald’s distinctive yellow arches as golden helps to enhance people’s valuation of the brand (e.g., Graham, 2016).

22 Ernest Hemingway also writes of the metallic taste of raw oysters: “As I ate the oysters with their strong taste of the sea and their faint metallic taste that the cold white wine washed away, leaving only the sea taste and the succulent texture, and as I drank their cold liquid from each shell and washed it down with the crisp taste of the wine, I lost the empty feeling and began to be happy, and to make plans” (Hemingway, *A Moveable Feast,* 1964, p. 18).

23 Glindemann et al. (2006) suggest that “Biologists may interpret the distinctive and sensitive human smell for the (blood-)iron–skin odour as an evolutionary advantage that is much older than human use of metal tools: Early humans and their animal ancestors could probably like ‘blood-trailing’ dogs track and locate wounded prey or tribal comrades by the metallic ‘blood scent’ (of 1-octen-3-one etc.)” (p. 7007).

24 Although the story may have been different when it comes to Dracula (see Griffiths, 2014).

25 Relevant in this regard, Glindemann et al. (2006) note that people are “perplexed” by the metal odour given off by iron metal objects when touched.
